# Integrating Music Therapy and Nursing for Neonatal Procedural Support Using a Pacifier Activated Lullaby Device

**DOI:** 10.1097/AJN.0000000000000342

**Published:** 2026-07-23

**Authors:** Cassi Crouse, Margaret Gettis, Regena Spratling

**Affiliations:** **Cassi Crouse** is lead music therapist and **Margaret Gettis** and **Regena Spratling** are nurse scientists at Children's Healthcare of Atlanta in Atlanta, GA. Contact author: Cassi Crouse, catherine.crouse@choa.org. The authors have disclosed no potential conflicts of interest, financial or otherwise.

**Keywords:** music therapy, neonatal intensive care unit, pacifier activated lullaby device, procedural support, therapeutic music intervention

## Abstract

**Background:**

Premature neonates may require hospitalization in the neonatal intensive care unit (NICU), where they receive life-sustaining medical treatment. Care often involves the need for painful, invasive medical procedures. Music therapists working with nurses in pediatric health care settings can provide procedural support to patients via therapeutic music interventions.

**Purpose:**

The purpose of this evidence-based practice pilot initiative was to examine the impact of a pacifier activated lullaby (PAL) device intervention, facilitated by a NICU music therapist (NICU-MT) in collaboration with bedside nurses, on neonates undergoing retinopathy of prematurity (ROP) exams in the NICU.

**Methods:**

The NICU-MT facilitated the PAL device intervention for neonates (N = 25) following ROP exams. Data were collected before the procedure; at the end of the procedure; and at one, three, and five minutes of PAL device use. Measurements to assess infant pain and stress before, during, and after the procedure included vital signs and pain scores.

**Results:**

Findings reflected improvements in vital signs and pain scores following the five-minute PAL intervention, with measurements nearing preprocedure baseline averages, despite noteworthy end-of-procedure averages (including increased heart rate, decreased arterial oxygen saturation, and elevated pain scores). Outcomes from the pilot suggest that the PAL device may encourage a return to preprocedure baseline levels following ROP exams.

**Conclusion:**

NICU-MTs and nurses can collaboratively provide procedural support using a therapeutic music intervention such as the PAL device to comfort neonates during painful, invasive procedures.

**Figure:**
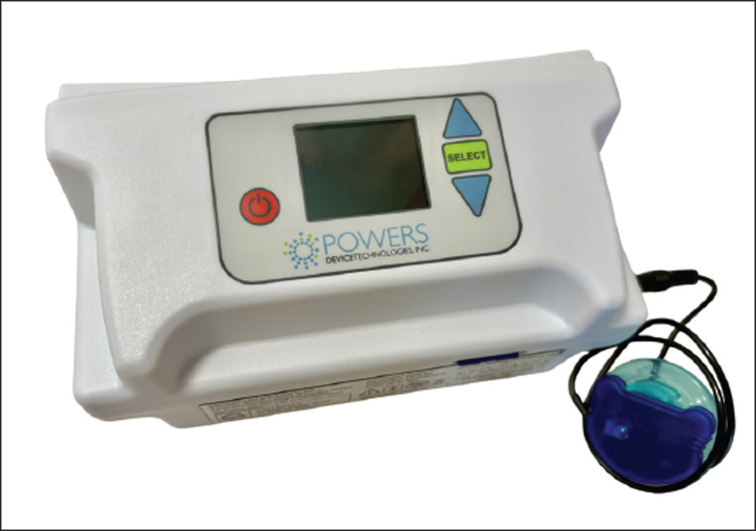
A pacifier activated lullaby device. Photo by Cassi Crouse.

Premature neonates may require hospitalization in the neonatal intensive care unit (NICU) for critical treatment and life-sustaining care. The intensive care environment is often not conducive to important neurodevelopmental needs such as rest and regulation, even when developmental care is implemented on the unit. Frequent exposure to painful procedures and aversive stimuli can lead to overstimulation, which significantly impacts neurological growth and development in neonates.^1^ As neonates mature outside the protective environment of the womb, bedside nurses, health care providers, and allied health clinicians must work collaboratively to support neuroprotective measures and developmental care needs during hospitalization.

**Available knowledge**. Invasive procedures are the leading cause of pain for neonates in the NICU.^2^ Ophthalmic exams are administered by pediatric ophthalmologists to screen for retinopathy of prematurity (ROP), a disease known to cause blindness. The exams are recurring, intrusive, and painful, with speculum insertion identified as the period of greatest discomfort.^3^-^5^ Significant physiological and behavioral changes are present when a neonate is in pain, and interventions aimed at reducing or relieving pain are important for developmentally supportive care, particularly during ophthalmic exams.^6^-^8^

Pharmacologic and nonpharmacologic interventions may be effective in mitigating pain with ROP exams. Pharmacologic interventions include topical anesthetics and oral analgesics.^3^,^5^,^7^,^9^-^11^ Nonpharmacologic interventions include utilization of breastfeeding^2^,^6^,^8^,^12^; breast milk^4^,^5^,^7^,^8^; glucose and sucrose^2^,^3^,^5^-^7^,^9^; massage and touch^13^; nonnutritive sucking^2^,^3^,^6^,^7^,^9^,^12^,^14^; tucking, swaddling, nesting, and containment^7^,^14^; music-based interventions^15^,^16^; and music therapy.^17^-^20^ Multisensory interventions, such as nonnutritive sucking, and combining pharmacologic and nonpharmacologic support strategies are the most efficacious approaches to promoting comfort and reducing pain during ROP exams.^9^,^10^,^15^,^21^,^22^

The use of music-based interventions is an accessible and familiar nonpharmacologic strategy implemented in the health care setting for patients of all ages, including neonates. Gao and colleagues examined the efficacy of routine care versus a combination of nonpharmacologic interventions, including music, to address procedural pain in infants in a NICU and determined that the combined interventions were efficacious and safe for reducing pain.^15^ A study exploring the combined use of recorded music and nonnutritive sucking versus standard care for neonatal pain management found notable improvements in pain scores and vital signs in the intervention group compared to the standard care group.^23^

Music therapy—the use of music-based interventions within a therapeutic relationship by a certified music therapist—has been used in NICUs for many years. A growing body of research demonstrates its benefits for premature neonates and their families.^24^,^25^ Positive, meaningful, and developmentally appropriate auditory experiences provided via evidence-based music interventions and facilitated by NICU music therapists (NICU-MTs) support neonates experiencing pain, agitation, and stress during hospitalization.^26^,^27^

Yue and colleagues conducted a systematic review and meta-analysis of randomized controlled trials on the effectiveness of music therapy with premature neonates in the NICU.^25^ Results of the meta-analysis suggest music therapy is efficacious in improving heart rate and respiratory rate in such infants. Additionally, findings confirmed music therapy as a noninvasive nonpharmacologic procedural support for neonates in the NICU. In another study that aimed to determine the effects of music therapy on vital signs, feeding, and sleep, a live music intervention was provided by a music therapist to neonates ages 32 weeks and older.^19^ The intervention positively influenced vital signs and sucking patterns. Corrigan and colleagues studied a music therapy intervention in which neonates recovering from ROP exams heard recordings of their mothers' singing and heartbeat.^17^ Outcomes suggested that music therapy can be a cost-effective nonpharmacologic procedural intervention for neonates that may promote a faster return to baseline.

Use of a pacifier activated lullaby (PAL) device is a standard of care intervention provided by NICU-MTs. The device encourages nonnutritive sucking by playing developmentally appropriate, soothing music when the infant sucks on the pacifier. Nonnutritive sucking enhances physiological stability, is easily implemented (although underutilized), and is likely a successful intervention for decreasing pain.^2^-^4^,^12^ Clinical studies using PAL devices demonstrate benefits such as improved feeding, behavioral state regulation, and pain relief.^24^ Whipple found that neonates who used a PAL device while undergoing heel sticks had more stable behavioral states and stress levels, thus facilitating return to a baseline behavioral state.^20^ Another study using a PAL device during IV line placement in the NICU found that neonates who used it had more stable vital signs and spent more time in a calm behavioral state.^18^ While the PAL intervention may soothe neonates during painful procedures, it is also beneficial during routine care to facilitate positive behavioral states (such as quiet alert, drowsy, active sleep, and deep sleep) and encourage self-soothing behavior.^18^,^20^

**Rationale**. Given the high prevalence of painful, invasive procedures in the NICU and the potential for pain to affect neurodevelopment, developmental support provided by clinicians is essential for neonates' neuroprotection. Integrating opportunities for neonates to use a PAL device during or after painful procedures can promote their coping, recovery, and return to baseline states.

**Project purpose**. The purpose of this evidence-based practice (EBP) pilot initiative was to examine the impact of a PAL device intervention, facilitated by a music therapist in collaboration with bedside nurses, on neonates undergoing ROP exams.

## METHODS

This EBP initiative occurred at a pediatric health care organization in the southeastern United States. The organization has three hospital campuses with over 600 beds and is situated in a large, urban area. The hospital campus where the pilot took place has a level III NICU with approximately 40 beds. The NICU has a full-time NICU-MT who provides clinical music therapy services to neonates and their families.

The NICU-MT led the pilot in collaboration with a nurse scientist. The two clinicians completed a literature review exploring pharmacologic and nonpharmacologic interventions used during ROP exams, including music therapy. They synthesized the literature and presented the findings to NICU staff. Given the benefits demonstrated in the literature regarding the use of nonnutritive sucking and music therapy, and the unit's access to a full-time NICU-MT as well as a PAL device, the PAL intervention was selected as a cost-effective strategy to provide comfort to infants following ROP exams. An interdisciplinary neonatal team was formed to collaborate with the NICU-MT to ensure successful implementation of the project.

The pilot initiative occurred over a three-month period, March through May 2024, and included neonates admitted to the NICU during that period. The target population was neonates undergoing ROP exams who were medically stable and developmentally able to engage in nonnutritive sucking. Exclusionary criteria included requiring high levels of respiratory support that made pacifier use unsafe, requiring intubation, and/or being deemed medically unstable at the time of the ROP exam.

**Intervention**. On designated ROP exam days, the NICU-MT received a list of patients set for the exam and the anticipated schedule from the neonatal team. The NICU-MT then reviewed patient medical charts to determine medical status, level of respiratory support, and ability to safely use a pacifier. Following the chart review, the NICU-MT prepared a patient census and engaged with bedside RNs to confirm whether the neonates were currently stable and appropriate candidates for the PAL intervention. If an infant was deemed eligible, the NICU-MT and bedside RN introduced the intervention to the infant's family caregivers prior to the exam and answered any questions. Families had the option to decline participation in the pilot prior to exam initiation.

The NICU-MT and a data collection nurse followed the ROP exam team as they conducted the exams. The NICU-MT prepared the PAL device, attached an appropriately sized pacifier (Soothie or Wee Soothie) to the device's sensor, and stood by to initiate the intervention at the conclusion of each exam.

The usual standard care practices to promote comfort during ROP exams, such as holding, positioning, and swaddling, continued as normal. Family caregivers did not hold their infant during the exam, as the infant was supported by staff and space at the bedside was limited. The bedside RN provided emotional support to the family caregivers as needed or remained to support the exam team. When the exam concluded, the bedside RN notified the family, allowing them to return to the bedside and provide comfort to their infant. Family caregivers were present at the bedside for most of the PAL interventions.

Once the exam was complete and the physician exited the bedspace, the NICU-MT offered the neonate a pacifier attached to the PAL device sensor for a duration of five minutes. The prerecorded music provided with the PAL device was used for consistency. The data collection nurse informed the NICU-MT when the five-minute interval was over. The NICU-MT then turned off the PAL device and removed the sensor from the pacifier, allowing the infant to continue using the pacifier. The NICU-MT cleaned the device, debriefed the bedside nurse, and moved on to the next exam with the data collection nurse.

After all the exams were completed, the NICU-MT documented the intervention in each infant's electronic health record using a standard NICU music therapy session note.

**Measures**. In the literature, outcome measures of the use of music therapy in the NICU typically comprise physiological measures (most commonly heart rate and oxygen saturation), behavioral state, and pain.^16^,^18^-^20^,^23^,^25^ In this initiative, measures included heart rate, arterial oxygen saturation (SaO_2_), and respiratory rate, which were observed via the bedside monitor; and pain scores, which were generated using the Neonatal Pain, Agitation, and Sedation Scale (N-PASS), a validated assessment tool comprising five indicators: crying/irritability, behavior/state, facial expression, extremities/tone, and vital signs.^28^

Data were collected before the ROP exam; at the end of the exam; and at one, three, and five minutes of PAL device use. The time intervals selected allowed for observation of potential trends in the data. The data collection nurse recorded the data on a form designed by the NICU-MT and later transferred the data to Research Electronic Data Capture (REDCap), a secure database used by health care professionals to manage data. N-PASS pain scores were calculated in REDCap to avoid human error. Reports offered by REDCap were exported for data analysis upon the conclusion of the pilot.

**Analysis**. Deidentified data from REDCap reports were exported to Excel for further analysis. Data were analyzed using descriptive statistics for each outcome measure at the five intervals. Results were then used to create tables and figures and were sorted by outcome measure. At certain time intervals, data were unable to be collected for all participants. Physiological data was the outcome area with the most missing information.

**Ethical considerations**. The health care organization's institutional review board determined that this initiative was exempt from oversight.

## RESULTS

A total of 25 neonates were included in the analysis; however, there were missing data for some neonates due to absent information on patient monitors. Nineteen neonates were male (76%) and six were female (24%). Six neonates utilized the PAL device multiple times over the three-month period. The neonates' gestational ages ranged from 22.14 to 34 weeks, averaging 24.92 weeks. Postmenstrual ages ranged from 31.43 to 55.43 weeks and averaged 42.43 weeks. Chronological ages ranged from 7.43 to 31.86 weeks, with an average of 17.51 weeks. The average weight of the neonates was 2.88 kg.

The number of neonates receiving the PAL intervention ranged from zero to five per ROP exam day. There was one week when no neonates receiving ROP exams were eligible to use the PAL device. The ROP exams varied in length depending on factors such as infant response (higher agitation or distress led to longer exams) and depth of the clinical exam.

**Figure 1.**
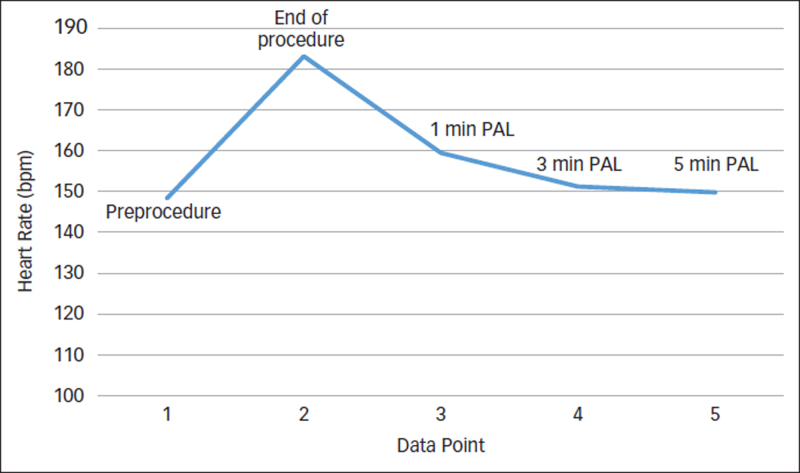
Mean Heart Rate at Project Intervals

**Vital signs**. The mean baseline heart rate prior to the ROP exam was 148.32 beats per minute (bpm). The mean heart rate at the end of the exam was 183.13 bpm. Mean heart rate during use of the PAL device was 159.46 bpm at one minute, 151.21 bpm at three minutes, and 149.79 bpm at five minutes (see Figure 1). Mean infant heart rate returned close to baseline during use of the PAL device, following the increase in mean heart rate during the ROP exam (see Table 1).

PAL = pacifier activated lullaby.

**Table 1.** Vital Signs and N-PASS Pain Scores at Project Intervals

	Preprocedure	End of Procedure	1 Min PAL	3 Min PAL	5 Min PAL
Heart rate (beats per minute)					
No. of neonates	25	24	24	24	24
Mean	148.32	183.125	159.458	151.208	149.792
SD	17.521	16.448	16.767	13.368	15.079
Min.	120.00	156.00	125.00	126.00	125.00
Max.	180.00	219.00	191.00	176.00	174.00
Arterial oxygen saturation (%)					
No. of neonates	25	23	25	25	24
Mean	95.040	88.522	97.200	97.120	97.00
SD	3.984	8.479	3.055	2.698	2.638
Min.	86	68	88	92	92
Max.	100	100	100	100	100
Respiratory rate (breaths per minute)					
No. of neonates	24	21	23	22	23
Mean	50.125	63.333	63.130	61.182	56.261
SD	14.513	17.494	18.046	14.809	12.304
Min.	30	39.000	40.000	45.000	40.000
Max.	90	100.000	100.000	92.000	82.000
N-PASS pain score					
No. of neonates	25	25	25	25	24
Mean	0.470	4.920	0.760	0.600	0.208
SD	0.918	1.605	0.831	0.913	0.509
Min.	0.000	2.000	0.000	0.000	0.000
Max.	4.000	8.000	3.000	3.000	2.000

N-PASS = Neonatal Pain, Agitation, and Sedation Scale; PAL = pacifier activated lullaby.

**Figure 2.**
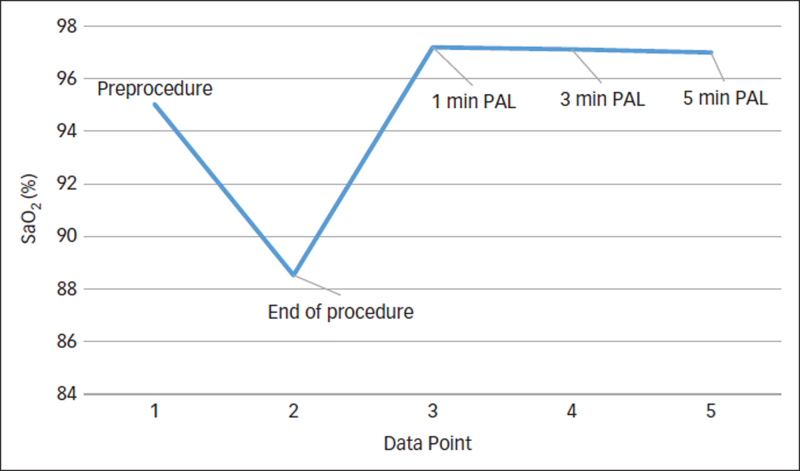
Mean SaO_2_ at Project Intervals

The mean SaO_2_ level prior to the ROP exam was 95.04%. The mean SaO_2_ level at the end of the exam was 88.52%. Mean SaO_2_ levels during use of the PAL device were 97.2% at one minute, 97.12% at three minutes, and 97% at five minutes. Following the decrease in mean SaO_2_ levels at the end of the ROP exam, there was a slight improvement in mean SaO_2_ levels compared to baseline during the PAL intervention (see Figure 2).

PAL = pacifier activated lullaby; SaO_2_ = arterial oxygen saturation.

**Figure 3.**
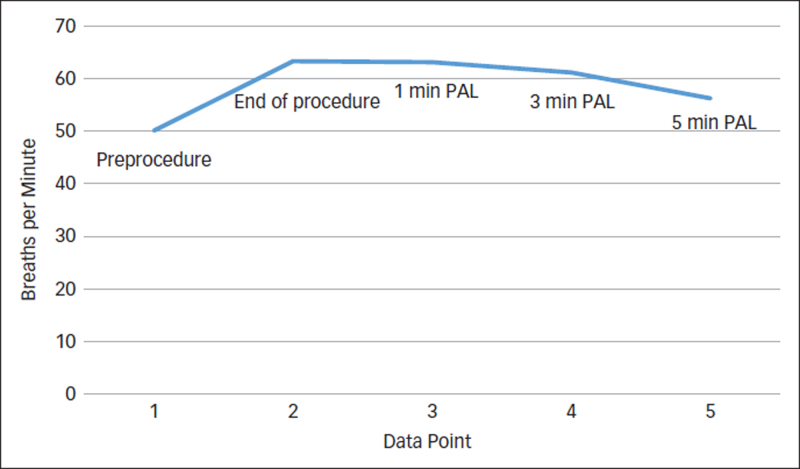
Mean Respiratory Rate at Project Intervals

The mean respiratory rate prior to the ROP exam was 50.13 breaths per minute. At the end of the exam, the mean respiratory rate was 63.33 breaths per minute. Mean breaths per minute during PAL device use were 63.13 at one minute, 61.18 at three minutes, and 56.26 at five minutes. Respiratory rates increased with the ROP exam and required a longer time to recover than heart rate and oxygen saturation (see Figure 3).

PAL = pacifier activated lullaby.

**Figure 4.**
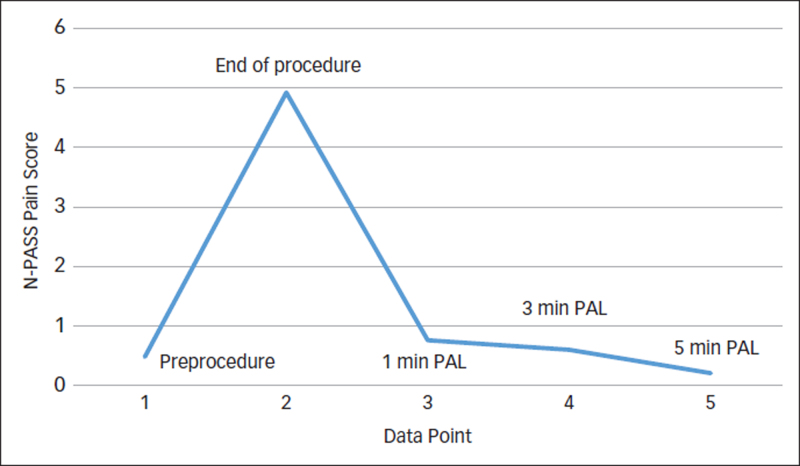
Mean N-PASS Pain Scores at Project Intervals

**N-PASS pain scores**. The mean preprocedure N-PASS pain score was 0.47, indicating a comfortable baseline with minimal to no pain (see Figure 4). The mean N-PASS pain score at the end of the exam was 4.92, with the increased score suggesting the neonates were experiencing increased levels of pain. Once the PAL intervention began, N-PASS pain scores dropped quickly: at one minute, the mean score was 0.76; three minutes, 0.6; and five minutes, 0.21. At the end of the PAL intervention, the mean N-PASS pain score was lower than the preprocedure score.

N-PASS = Neonatal Pain, Agitation, and Sedation Scale; PAL = pacifier activated lullaby.

## DISCUSSION

The outcomes of this pilot initiative indicate that music therapy–facilitated procedural support utilizing the PAL device following ROP exams can be effective in mitigating neonates' pain and providing comfort. The outcomes suggest that the PAL device may be used as a therapeutic tool when providing music therapy–facilitated procedural support to neonates to reduce distress following painful, invasive procedures.

**Implications for practice**. NICU-MTs receive specialized education and training that informs their clinical approach to providing therapeutic music interventions in the NICU, ensuring care is safe, effective, and supported by evidence. They work collaboratively with bedside nurses to provide developmentally supportive care to neonates and their families. Bedside RNs can be advocates and refer patients receiving painful, invasive procedures to music therapy services. Collectively, NICU-MTs and RNs can identify additional opportunities to support hospitalized neonates and their families. Additionally, NICU-MTs can engage in ongoing advocacy and provide education regarding the NICU-MT role and the scope of music therapy services within the NICU.

Health care organizations should consider increasing music therapist staffing, ensuring all patients and families have access to music therapy services. Operationalizing music therapy positions and program funding ensures program health and longevity, access to training and education, and availability of resources and equipment.^29^,^30^ By increasing music therapist staffing, nurses and all health care providers have opportunities to improve individualized care, implement EBP initiatives, and engage in best practices. In addition, ongoing research on the use of music therapy interventions for patients undergoing painful or invasive procedures can progress.

**Limitations**. Limitations of this initiative included a small sample size, a single-site NICU, time constraints, and limited access to funding and resources. Another limitation was the inability to use the PAL device during other painful procedures. Additionally, due to staffing constraints, only one NICU-MT was available; therefore, not every eligible infant received the PAL intervention. As the NICU-MT provided the intervention to one infant, the physician often moved to a new patient before the intervention and/or data collection concluded for the previous patient. Lastly, the NICU-MT is not a position all NICUs have. While it is ultimately recommended that a NICU-MT implement the PAL device as a therapeutic tool collaboratively with bedside nurses, leadership in NICUs can consult with NICU-MTs to receive training on utilizing the PAL device. This presents an opportunity for bedside nurses to introduce an additional comfort offering to infants experiencing pain and discomfort, which also serves as a reassuring measure for family caregivers.

## CONCLUSIONS

Music therapy in the NICU is grounded in developmental support and family-centered care and can be utilized to support neonates and their families during hospitalization.^24^,^26^ NICU-MTs and nurses can collaborate to provide nonpharmacologic procedural support using a therapeutic music intervention such as the PAL device to reduce stress and discomfort and mitigate pain during painful or invasive procedures.
